# Experiments and Observations to Investigate the Composition of James's Powder

**Published:** 1792

**Authors:** George Pearson


XIV. *Experiments and Obſervations to inveſtigate the Compoſition of James's Powder.* *By* George Pearſon, *M. D. F. R. S.*—Vide *Philoſophical Tranſactions of the Royal Society of London, Vol.* LXXXI. *for the Year* 1791. *Part* II. 4to. London, 1791.

ALTHOUGH the powder, which is the ſubject of the paper before us, has for more than thirty years been very extenſively uſed in the cure of continued fevers, the Public have not been accurately informed of the particular nature of this ſubſtance. It was originally a patent medicine; but it is well known that it cannot be prepared by following the directions of the ſpecification in the Court of Chancery. Dr. Pearſon has, therefore, been induced to make it the ſubject of chemical inveſtigation, and by a great number of judicious experiments, ſynthetical as well as analytical, has attempted to aſcertain the nature and manner of preparing this medicine.

He begins with deſcribing its ſenſible properties. Some parcels of this preparation, he obſerves, are white, but in general it has a yellowiſh caſt; and this ſhade is more evident in

ſome ſpecimens than in others. It is ſaid, that this powder cannot, in general, be made at different times of preciſely the ſame ſhade of yellow or degree of whiteneſs. Sometimes with the aid of a lens a few very ſmall ſhining *ſpicula* are ſeen mixed with powder. When preſſed between the fingers it feels ſmooth, with ſome rather rough particles, and it is gritty in the mouth. Moſt parcels at firſt are taſteleſs; but in about a minute there is a ſlight braſſy taſte. It is perfectly inodorous.

Dr. Pearſon next ſpeaks of its ſpecific gravity. He obſerves that it feels much heavier than any of the common earths and ſtones in a pulverized ſtate. One of the phials, nearly full, in which it is ſold, reckoned to hold a quantity equal to twelve packets, or 480 grains, contained 470 grains troy weight of James's Powder. This phial, filled with diſtilled water to the ſame height that it had been by the powder, was found to contain nearly four drachm-meaſures, or about 240 grains, of this liquid.

In another experiment made in a different manner, for the purpoſe of aſcertaining the ſpecific gravity of this powder, the quantity which nearly filled a phial weighed 437 grains; and upon filling the ſame phial, to the ſame height, with diftilled water, the temperature of which was 65°, the water weighed 250,2 grains. The reafon of the variation in thefe refults, in making ufe of different parcels of this medicine, our author thinks, will be obvious, from the account he gives of its preparation, and the great difficulty of determining, with accuracy, the fpecific gravity of powders.

After defcribing the effects of fire on this fubftance, and obferving that the experiments made for this purpofe indicated the prefence of a metallic calx, a part at leaft of which was that of antimony, mixed with earthy matter, he proceeds to experiments with different menftrua, viz. water, the acetous, nitrous, and marine acids.

From his experiments on James's powder with the firft of thefe menftrua, viz. water, he thinks we may conclude,

1. That the whole, or a part of it, is foluble, or at leaft may be fufpended, in about 2000 times its weight of pure water, cold; and in about half this quantity of boiling water.

2. That this folution contains calcareous earth united to an acid, or fome other fubftance, from which it cannot be difunited by cauftic or mild fixed alkalies.

3. That

3. That this ſolution contains a metallic calx, a part of which at leaſt is that of antimony uncombined, or at leaſt not united to any acid with which it forms a compound ſoluble in water.

4. That the ſubſtance in a nitrous ſolution of the part of James's Powder that had been diſſolved in water, which was obſerved to precipitate lime from lime-water, and which percipitate was not ſoluble in a large quantity of vinegar, is, probably, phoſphoric acid from phoſphorated lime decompoſed by nitrous acid.

The precipitation by muriated barytes and nitrated ſilver, which is deſcribed as having taken place in two of the experiments, could not, our author thinks, be from vitriolic and marine acids conſiſtently with the preceding experiments; and he could not have conjectured what was the ingredient in James's Powder which occaſions it, if he had not found, that muriated barytes is not only a teſt of vitriolic but of phoſphoric acid united to lime and alkalies; and that the acid of phoſphorus will alſo produce a turbid appearance with nitrated ſilver.

The preſence of calx of iron was detected in theſe experiments, but in ſo ſmall a quantity that our author is inclined to conſider it only as an accidental ſubſtance.

His experiments with the acetous acid indicate the ſame kind of ſubſtances as the experiments with water, namely, calcareous earth in a combined ſtate; phoſphoric acid ; calx of antimony, and calx of iron. It appears alſo from theſe experiments that James's Powder is either wholly or partially ſoluble in about 300 times its weight of concentrated acetous acid.

It appears from the experiments with nitrous acid, that this menſtruum, by two affuſions, in a large proportion, aided by trituration, digeſtion, and heat, diſſolved $\frac{108}{240}$ of James's powder that had been expoſed to the action of water and acetous acid ; but our author obſerves, that from the ſmallneſs of the quantity which was contained in the nitrous acid the ſecond time it was applied, and from its being principally calcined antimony, not more than two of the ſix grains afforded by the ſolution, perhaps, ſhould be conſidered as *diſſolved*, for the reſt may be ſuppoſed to have been merely *ſuſpended.*

The firſt ſolution alſo in this menſtruum, it ſeems, was not filtered, and the acid was conſiderably redundant, and there were found in it ſeveral grains of calcined antimony. The real quantity *diſſolved* might, therefore, our author thinks, probably be eight grains leſs than the above 108 ſtated. According to this mode of calculation, the proportion of the ſoluble part of James's Powder in nitrous acid is $\frac{100}{240}$, or about $\frac{41}{100}$.

2. The whole of this ſoluble part, except a little calx of antimony, he thinks, is deciſively phoſphoric acid and calcareous earth: which two ſubſtances, he obſerves, may reaſonably be ſuppoſed, from theſe experiments, to have been united together, and to have been in the ſtate of phoſphorated lime in this powder. Conſequently the proportion of this phoſphorated lime, conſidered as the ſoluble part of James's Powder in the experiments in queſtion with nitrous acid, appears to be 40 *per cent.* making a deduction of 1 *per cent.* for the antimonial calx contained in the nitrous acid, in theſe experiments. He ſuſpects, however, that the powder which reſiſted ſolution in this menſtruum may contain more phoſphorated lime; and this conſideration prevents him aſſigning at preſent the above 40 *per cent.* as the whole quantity of it in James's Powder. It cannot, however, he thinks, be a ſmaller proportion.

He does not reckon the calx of iron in theſe calculations, becauſe it is in too ſmall a quantity, and is apparently only to be looked upon as an accidental extraneous ſubſtance. He ſuppoſes

 too, too, that the water and acetous acid applied to the James's Powder uſed in theſe experiments, carried off a proportion of its ingredients equal to that in the remaining powder.

From the experiments the author next relates, we learn, that by repeatedly digeſting and boiling in marine acid, and in *aqua regia*, that part of James's Powder which reſiſted ſolution in nitrous acid, which was $\frac{108}{240}$, 77 grains were carried off by theſe menſtrua; but conſidering the ſmall proportion contained in theſe acids after the two firſt affuſions, which afforded 57,15 grains, and ſuppoſing the calx to be neither increaſed nor diminiſhed in weight by the acids, the real quantity of ſoluble and fuſible calx of antimony, he thinks, may be ſtated to be that of the Algaroth powder which he obtained in theſe experiments; for the other kind of antimonial calx procured by ſubſequent affuſions was probably only *ſuſpended.* It appears, therefore, that 240 grains of James's Powder, afforded, by theſe experiments with marine acid, 57,15 grains of Algaroth powder, and 19,85 grains of a leſs ſoluble and more difficultly fuſible calx of antimony, with a ſmall proportion of phoſphorated lime. The reſiduum, amounting to 55 grains, was next examined; from his experiments; ments, however, on this infoluble matter, he could only conclude that it contained calx of antimony; but as to the proportion of this calx, and the other fubftance with which it is joined, he conjectures that it may be about half the quantity of the infoluble powder; and that the other half is antimonial calx and phofphorated lime, fo highly calcined and vitrified together as to refift folution in acid menftrua, decompofition by charcoal, and fufion with fixed alkalies, but not by phofphoric acid.

Our author obferves, that he fhould not have been fatisfied with here terminating this analyfis without enquiring further into the nature of this infoluble matter; but he difcontinued this analytic inveftigation in order to derive light from fynthetic experiments.

His experiments on this infoluble matter feem to fhow, that the proportion of antimonial calx is not fo great as might have been affigned from the experiments with nitrous acid, marine acid, and *aqua regia*.

The fubftances and proportions of them, obtained from 240 grains of James's Powder, were as follow:

| | Grains. |
|---|---|
| Phofphorated lime, with a little antimonial calx, - - | 100, |
| Algaroth powder, - - | 57,15 |
| Infoluble antimonial calx, with a little phofphorated lime, - - | 19,85 |
| The fame infoluble calx, with, probably, a little phofporated lime, - | 55, |
| Wafte, - - | 8, |
| | 240,0 |

As it might be objected, that conclufions drawn concerning the nature of calces might be erroneous if nitrous acid had been applied previoufly to fubftances containing them, our author made an experiment with marine acid applied to James's Powder, which had not been expofed to the action of nitrous acid, or any other menftruum. From this experiment he thinks it may, perhaps, be fairly concluded that the properties of the calx in James's Powder are not altered by nitrous acid to affect its folubility in marine acid.

To know whether James's Powder contained any fubftance that could be decompofed by mild fixed alkalies, 100 grains of James's Powder were boiled in fix ounces of water, with fifty grains grains of mild alkali of tartar, for three hours, and then the remaining liquid was filtered, and evaporated to dryneſs; but the matter left after evaporation was nothing but the alkali uſed in the experiment, with a little of the powder itſelf.

The reſult, we are told, was the ſame on making the experiment with cryſtallized mineral alkali inſtead of alkali of tartar.

Although the inability to prepare James's Powder would not prove the above concluſions, with reſpect to its compoſition, to be erroneous, the being able to compoſe a ſubſtance poſſeſſing all the ſame properties as James's Powder, by uniting or mixing together the ſubſtances ſhewn by the preſent analyſis to enter into its compoſition, would afford, our author thinks, all the proof and demonſtration which can be had from chemiſtry.

His analyſis, he obſerves, ſhewed no eſſential ingredients of James's Powder but antimonial calces, phoſphoric acid, and calcareous earth, which two laſt ſubſtances appeared to be united together; but it would, he contends, have been vain and unneceſſary labour to have attempted to make this powder by mixtures of any of the commonly known calces of antimony and phoſ-

phorated phorated lime; becaufe none of them, from their well-known qualities, could form a powder of the fame colour and fpecific gravity as James's Powder, and like it partially foluble in acids. From his experiments, however, the probability, he obferves, was evident, that this fubftance might be made by calcining together antimony and bone-afhes; which operation produces a powder called Lile's and Schawanberg's fever-powder; a preparation difcribed by Schröder * and other chemifts more than a century ago. The receipts for this preparation differ in the proportion of the antimony to the bone afhes, and in the ftate of the bone; fome directing bone fhavings to be previoufly boiled in water; others ordering them to be burnt to afhes before calcining them with antimony; while in other prefcriptions the bone fhavings are directed to be burnt with the antimony. According to a receipt in the poffeffion of Mr. Bromfeild, by which this powder was prepared forty-five years ago, and before any medicine was known by the name of James's Powder, two pounds of hart's-horn fhavings muft be boiled to diffolve

* Pharmacop. Med. Chymic. 8vo. Lug. Bat. 1672, p. 428.

all all the mucilage, and then, being dried, be calcined with one pound of crude antimony, till the ſmell of ſulphur ceaſes, and a light grey powder is produced. The ſame preſcription, we are told, was given to Mr. Willis, about forty years ago, by Dr. John Eaton, with the material addition, however, of ordering the calcined mixture to be expoſed to a great heat in a cloſe veſſel to render it white. Mr. Turner, it ſeems, made this powder above thirty years ago by calcining together equal weights of burnt hart's horn and antimony in an open veſſel, till all the ſulphur was driven off, and the mixture was of a light grey colour. He likewiſe was acquainted with the fact, that by a ſufficient degree of fire in a cloſe veſſel this cineritious powder turned *white* *. Mr. Turner alſo prepared this powder with a pound and a half of hart's-horn ſhavings

* Dr. Pearſon thinks it probable that this powder was made for ſeveral years with merely the heat neceſſary to carry off the ſulphur and calcine the bone, in an open veſſel over a charcoal fire in a common grate, and conſequently it was of a light clay or aſh colour. In this manner, Mr. Bromfeild told him, he prepared Schawanberg's Powder 46 or 47 years ago. Its property of turning *white* in a greater degree of fire appears to have been a ſubſequent diſcovery.

and and a pound of antimony, as well as with ſmaller proportions of bone. Schröder preſcribes equal weights of antimony and calcined hart's horn; and Poterius and Michaëlis, as quoted by Frederic Hoffman, merely order the calcination of theſe two ſubſtances together (aſſigning no proportion), in a reverberatory fire for ſeveral days. In the London Pharmacopœia of 1788, this powder is called *Pulvis antimonialis*; and it is directed to be prepared by calcining together equal weights of hart's-horn ſhavings and antimony.

Our author obſerves that powders made from various proportions of antimony and bone aſhes, after ſolution in nitrous acid, left a reſiduum of antimonial calx much leſs or greater in quantity than James's Powder did by the ſame menſtruum, except two of Mr. Turner's proportions, viz. two parts of antimony and one of calcined bone, and equal weights of bone ſhavings and antimony. The quantity of this calx was, however, greater, it ſeems, in the powder from the former of theſe two laſt proportions than the latter of them; which latter correſponded ſometimes exactly, and always nearly, with the weight of the calx from a given weight of James's Powder. This calx afforded alſo, we we are told, the ſame proportion of Algaroth powder as the calx in James's Powder; and the inſoluble part of the calx afforded metallic grains like thoſe from the inſoluble part of the calx in that powder.

Dr. Pearſon found then an exact correſpondence between what he conſiders to be the eſſential and peculiar properties of James's Powder, and the properties of a powder made by uniting or mixing together the ingredients of James's Powder found by analyſis. But, in order to ſhow the identity or difference of the qualities of theſe two ſubſtances, he made comparative obſervations on them, and repeated his analytic experiments on James's Powder with the preparation made by calcining together equal weights of bone ſhavings and antimony, in an open veſſel, to carry off the ſulphur, and then in cloſe veſſels, applying ſuch a degree of fire as to render them white, that is, on the ſame preparation as the *Pulvis antimonialis* of the London Pharmacopœia.

Firſt, he compared, more particularly, the ſenſible qualities of ſeveral different ſpecimens of James's Powder with various parcels of the *Pulvis antimonialis* made by different chemiſts. All of theſe, he obſerves, would be called white powders, powders, but not two of them were ſo in the ſame degree. Moſt of the papers of the *Pulvis antimonialis* were whiter than thoſe of James's Powder; but others were of a very light ſtone colour, and ſome had a ſhade of yellow, ſo as to reſemble very exactly James's Powder; but all the parcels of James's Powder had either a ſhade of yellow or of ſtone colour, and none were perfectly white, or ſo white as ſome ſpecimens of the *Pulvis antimonialis*. Some of the parcels of James's Powder and of the *Pulvis antimonialis* taſted braſſy; and other ſpecimens of both powders had no taſte. All of theſe powders were gritty. Moſt of the parcels of the *Pulvis antimonialis* were a little ſpecifically heavier than thoſe of James's Powder. The ſpecific gravity of both powders was increaſed by expoſing them to ſuch a degree of fire as brought them into almoſt a ſemi-vitrified ſtate; and on the contrary, the ſpecific gravity of the *Pulvis antimonialis* was leſs than it is in its uſual ſtate, when made in ſuch a degree of fire that the mixture preſerves the powdery form.

The experiments with water on the *Pulvis antimonialis* produced, we are told, the ſame kind of appearances, but more ſlightly than thoſe with James's Powder; for the hot ſolution of the former grew lefs milky on cooling than that of the latter, and on evaporation to drynefs lefs fediment was found of the folution of *Pulvis antimonialis* than after that of James's Powder.

The experiments with acetous acid on the *Pulvis antimonialis* fhewed that this menftruum diffolved fometimes a greater, and fometimes a fmaller proportion of it than of James's Powder; and the diffolved matter was found to be antimonial calx, phofphorated lime, and calx of iron, and no other fubftance.

The proportion of foluble matter in nitrous acid was the fame, it is obferved, or nearly fo, of the *Pulvis antimonialis* as that of James's Powder; and this diffolved matter was phofphoric acid, calcareous earth, with a little antimonial calx, and a minute portion of calx of iron, as exactly as could be expected from the nature of the fubftances and the experiments, in the fame proportion as thofe in James's Powder.

The Algaroth powder, which our author obtained by means of folution of the *Pulvis antimonialis* in marine acid, was in the fame proportion, as nearly as could reafonably be expected from the nature of the experiments, as that obtained from James's Powder. And the part that refifted refifted folution in this menftruum was partially reducible to a metallic form, and had otherwife the fame properties, as far as difcovered, as the infoluble part of James Powder.

Dr. Pearfon, having now formed a powder poffeffed of properties fimilar in kind to every one of thofe afcertained in James's Powder, with fcarcely any difference in the degree of them, contends, that, if it be thought that among thefe properties are thofe which are effential and peculiar ones of James's Powder, the conclufion that thefe two are the fame kind of things muft be admitted to be juft. The nature of one of the ingredients of James's Powder, viz. the irreducible part of the infoluble matter, he confeffes, is not fully elucidated by his fynthetic experiments; but in fo far as they fhow, that this part equally exifts in the powder formed by calcining together antimony and bone, which is concluded to be James's Powder, the objection againft the conclufion with refpect to the identity of the two fubftances, on the ground of this inconfiderable part of James's Powder not being well underftood, muft, he thinks, be of little weight.

We come now to the fynthetic experiments which our author made with the view of confirming firming or invalidating the conclusions drawn from the above analysis, with respect to the ingredients and proportions of them in James's Powder.

From one of these experiments (the 3d), which consisted in calcining together equal weights of antimony and hart's-horn shavings, it appears that the calcination of antimony with bone ashes is much more speedy than when by itself; and from another of them (the 5th) we learn what degree of fire is necessary to render the antimony calcined with bone of a white colour; and that this whiteness does not depend on the air, but on the fire. We shall here give the whole of this experiment.

" *(a)* 1500 grains of the calcined mixture

" of antimony and bone, Exp. 3. were kept

" red hot in a close vessel for half an hour. On

" cooling, I found the powder changed from

" a cineritious or clay colour to a whitish colour

" with a shade of yellow. The sides of the

" crucible were not glazed. The pyrometer in

" the middle of the powder had contracted to

" 40°. This powder was much inferior in

" whiteness to James's Powder, being much

" yellower.

" *(b)* Another parcel of the same powder,

" Exp. 3. was exposed in the same manner,

" but to a greater degree of fire, in which the

" crucible was almoſt white hot for half an hour.

" After cooling, the powder was found changed

" to a looſely cohering, ſnow-white, heavy maſs,

" and the ſides of the crucible were covered

" with a yellow glaze. This maſs, which was

" eaſily detached from the veſſel, was found

" covered with a yellow vitreous coat over the

" whole ſurface of it that had been in contact

" with the crucible. In the white ſolid, on

" breaking it, many argentine *ſpicula* were ſeen.

" The pyrometer uſed in all theſe experiments

" indicated 71°.

" (c) 1500 grains of the ſame parcel, Exp. 3.

" were expoſed in an open crucible to the fire

" of a melting furnace; no fumes aroſe till the

" crucible began to be almoſt white hot. Af-

" ter inverting another crucible, with a ſmall

" hole in its bottom, the fumes continued to

" aſcend at times through the aperture for a

" quarter of an hour. The crucible was then

" taken out of the fire, and on cooling a whitiſh

" powder was found, but no glazing, and the

" pyrometer indicated 28°. On again expoſ-

" ing this crucible with one inverted over it in

" the melting furnace, but to a greater degree

" of fire, ſtill more fumes aroſe; but, on cool-

" ing, the charge was ſtill in the ſtate of a pow-

" der,

" der, though whiter than before; and the in-

" side of the inverted crucible was covered with

" silvery particles, and the hole of it was sur-

" rounded with argentine *spicula*, in a stellated

" form. The pyrometer indicated 39°. On

" reducing a little of this powder to a greater

" degree of fineness, it was as white as James's

" powder, with a yellowish cast like it, but in-

" ferior in whiteness to a specimen of *pulvis*

" *antimonialis*. This crucible, containing its

" charge, with a cover closely luted on it, was

" put again into the fire, which was raised much

" higher than before; and, after being exposed

" in it twenty minutes, the powder in the cruci-

" ble became a loosely cohering solid, as white

" as snow, with a vitreous yellow coat, as be-

" fore observed; the inside of the crucible was

" glazed and covered with *spicula*. The pyro-

" meter-piece in the middle of the powder was

" also covered with a yellow coat, but not glaz-

" ed, and it indicated 81°. This loosely coher-

" ing solid, being pulverized, afforded a *whiter*

" powder than James's powder.

(*d*) The crucible, with its charge (*b*), hav-
" ing a cover well luted on it, was again put
" into the furnace, and the fire raised to almost
" as great a degree as I was able. This intense

" heat was kept up above an hour. After cool-

" ing, a white hard ſolid maſs was found with-

" in the crucible. On breaking the veſſel, to

" detach from it the charge, this ſolid maſs was

" found as hard as marble, and to have received

" its figure from the crucible. Its ſurface was

" covered with a yellow vitreous coat, and the

" whole inſide of the veſſel had a beautiful

" gold-coloured glaze with many argentine

" *ſpicula*. The pyrometer piece in the middle

" of the charge was alſo covered with a fine

" yellow glaze, and indicated 166°. This

" ſolid, hard maſs weighed only twenty-one

" grains leſs than before the experiment,

" though the whole inſide of the crucible was

" glazed, and had ſhining *ſpicula* upon it. A

" piece of this hard maſs being pulverized, it

" afforded a whiter powder than James's pow-

" der is in general."

Our author thinks it will not be difficult, from his experiments, to give a probable reaſon for the James's powder being generally of a yellowiſh caſt, and for different parcels of it, as well as of the *pulvis antimonialis*, being generally of different degrees of whiteneſs and ſhades of yellow. The colour of this preparation is, however, he obſerves, a very delicate one. He once once directed a perſon to calcine together antimony and bone ſhavings, in the uſual manner, to that ſtate in which the white powder may be produced by a due degree of fire; but, inſtead of a ſnow-white maſs, he could not by any degree of fire obtain any colour but a dirty whitiſh or light ſtone colour; though repeated calcinations were employed. The reaſon of the failure was, that the earthen diſh had been broken during the calcination, and a few very ſmall pieces of it had ſcaled off, and being mixed with the powder occaſioned this diſappointment with reſpect to colour. The ſame diſappointment, it ſeems, has been alſo occaſioned by uſing a ruſty iron rod in calcining the mixture.

The bone-aſhes procured from the ſal ammoniac and ſpirit of hart's horn manufactories, frequently failed, he tells us, in producing a white powder; and ſo did ſometimes the bone-aſhes, called prepared hart's horn, ſold by druggiſts. Even after a fine white-coloured maſs had been made, if it was pulverized in an iron mortar that had extremely little calx upon its ſurface, or dirt, the powder was not white.

Dr. Pearſon has been told by ſome of the perſons who prepare the *pulvis antimonialis*, that the whiteſt colour is obtained by firſt boiling the bone ſhavings to diſſolve their mucilage, and then calcining them with antimony as above ſhewn. Mr. Lile's receipt, he obſerves, directs previous decoction of the hart's horn.

He thinks that the yellow coat and glaze on the ſides of the crucible and ſurface of the calcined mixture of bone and antimony, which, he obſerved in his experiments, ſhould be aſcribed rather to the fuſion of the clay of the crucible with the antimonial calx, than to the greater degree of fire in the part of the crucible in which it takes place; or than to the calx of iron and ſiliceous earth of the veſſel; becauſe the ſame yellow coat and glazing are produced on the Wedgwood pyrometer pieces, which are placed in the middle of the charge, and where the degree of heat cannot be ſo great as nearer the ſide of the crucible, and yet a ſnow-white maſs is produced between theſe clay pieces and the ſides of the crucible. This yellow coat, he obſerves, is one reaſon for the powder being of a ſhade of yellow in ſome ſpecimens.

From an experiment which he relates, he thinks it very probable, that no degree or duration of fire, applied in open or cloſe veſſels to antimony alone, can produce a calx of the ſame kind as that in James's powder: nor, perhaps, that ſuch a powder can be compoſed by fire applied, in cloſe veſſels, to calx of antimony mixed with calcined bone; but if antimony duly calcined be mixed with calcined bone, and expoſed to air, in a due degree of fire, for a ſufficient length of time, and then a ſtill greater degree of fire be applied to it in cloſe veſſels, ſuch a compound, he obſerves, may be formed as James's powder. The ſame experiment, he thinks, alſo proves, that the ſulphur in antimony is no ways neceſſary to the formation of this compound.

Dr. Pearſon concludes his paper with obſerving that, from the whole of his analytical experiments, it appears:

1. That James's powder conſiſts of phoſphoric acid, lime, and antimonial calx; with a minute quantity of calx of iron, which is to be conſidered as an accidental ſubſtance:

2. That either, theſe three eſſential ingredients are united with each other, forming a triple compound; or, phoſphorated lime is combined with the antimonial calx, compoſing a double compound in the proportion of about fifty-ſeven parts of calx and forty-three parts of phoſphorated lime:

3. That this antimonial calx is different from any other known calx of antimony in ſeveral of its chemical qualities. About three fourths of it are ſoluble in marine acid, and afford Algaroth powder; and the remainder is not ſoluble in this menſtruum, and is apparently vitrified:

And from the ſynthetic experiments he has related, he thinks, it appears, that by calcining together bone-aſhes, that is, phoſphorated lime, and antimony in a certain proportion, and afterwards expoſing the mixture to a white heat, a compound was formed conſiſting of antimonial calx and phoſphorated lime, in the ſame proportion, and poſſeſſing the ſame kind of chemical properties, as James's powder.

Beſides the preparation, which is more immediately the ſubject of this paper, Dr. Pearſon has examined another medicine ſold under the title of "James's powder for horſes, horned cattle, hounds, &c." It is a light clay-coloured, gritty, taſteleſs ſubſtance, in which are ſeen ſmall *ſpicula*; and it appears to him to be nothing more than James's powder for fevers, or Lile's powder, made by calcining antimony and bone-aſhes together in open veſſels.

